# Draft Genome Sequence of Streptococcus salivarius AGIRA0003, Isolated from Functional Gastrointestinal Disorder Duodenal Tissue

**DOI:** 10.1128/MRA.00758-21

**Published:** 2021-09-30

**Authors:** Emily C. Hoedt, Erin R. Shanahan, Simon Keely, Ayesha Shah, Grace L. Burns, Gerald J. Holtmann, Nicholas J. Talley, Mark Morrison

**Affiliations:** a Viruses, Infection, Immunity, Vaccine and Asthma (VIVA) Program, Hunter Medical Research Institute (HMRI), Newcastle, New South Wales, Australia; b NHMRC Centre of Research Excellence (CRE) in Digestive Health, HMRI, Newcastle, New South Wales, Australia; c School of Medicine and Public Health, University of Newcastle, New Lambton, New South Wales, Australia; d Department of Gastroenterology and Hepatology, Princess Alexandra Hospital, Woolloongabba, Queensland, Australia; e Faculty of Medicine, The University of Queensland, St Lucia, Queensland, Australia; f School of Life and Environmental Sciences, University of Sydney, Camperdown, New South Wales, Australia; g School of Biomedical Sciences and Pharmacy, University of Newcastle, Callaghan, Australia; h Faculty of Health and Behavioural Sciences, The University of Queensland, St Lucia, Queensland, Australia; i Diamantina Institute, Faculty of Medicine, University of Queensland, Woolloongabba, Australia; University of Rochester School of Medicine and Dentistry

## Abstract

Patients suffering functional dyspepsia symptoms have been shown to possess a greater relative abundance of Streptococcus compared to asymptomatic controls. Here, we describe the isolation and genomic features of a new Streptococcus isolate, from the duodenal tissue of a subject reporting dyspeptic symptoms, taxonomically assigned to Streptococcus salivarius and designated strain AGIRA0003.

## ANNOUNCEMENT

Unexplained gastrointestinal symptoms cause considerable morbidity and are one of the most common reasons for medical consultations throughout the world (1). After ruling out other causes for the symptoms, most patients are diagnosed with a functional gastrointestinal disorder (FGID), which are currently most commonly differentiated by subjective, patient-reported symptoms into upper (functional dyspepsia [FD]) and lower (irritable bowel syndrome [IBS]) manifestations (1, 2). We have found that there are changes in the relative abundance of key bacterial taxa, and bacterial load, on the duodenal mucosa of FD patients compared to non-FD control subjects (3), but the functional basis of this dysbiosis remains largely undefined. To that end, we are developing methods to support the capture of mucosa-associated microbiota using a novel *ex vivo* combination of microbial culture with (meta)genomic sequencing (4). Here, we report the recovery and draft genome sequence of a Streptococcus salivarius strain isolated from a biopsy specimen of duodenal tissue from a patient diagnosed with FD.

During endoscopy, biopsy specimens were collected from the second portion of the duodenum (D2) utilizing the Brisbane aseptic biopsy device (MTW, Germany) (5), which enables aseptic collection and prevents cross contamination by oral or luminal contents. The entire device was placed in a plastic bag filled with CO_2_ and transported on ice to the lab. The biopsy specimen was processed inside an anaerobic chamber (10% H_2_, 10% CO_2_, and 80% N_2_), placed into a prereduced, anoxic, sterilized solution of 30% (vol/vol) glycerol, and then stored at −80°C for later culture.

Biopsy tissue stored in the sterile, anaerobically prepared cryopreservative buffer was aseptically transferred within an anaerobic chamber to a 10 ml volume of anaerobically prepared brain heart infusion (BHI; Oxoid) broth with added hemin (10 μg/ml). Vitamin K (0.5 μg/ml) was added as a sterile solution to the medium postautoclaving. The culture tubes were then incubated at 37°C overnight and returned to the anaerobic chamber, and a 0.1-ml volume of the resulting cultured bacteria was taken and used to stage a 10-fold serial dilution, with 0.1-ml aliquots from each dilution plated onto BHI agar medium with added hemin. Following the incubation of these plates within the anaerobic chamber at 37°C, discrete colonies were sampled with a sterile, disposable inoculation loop and streaked for single colonies onto fresh agar plates as described above. Following visual and microscopic purity checks, a single colony was inoculated into fresh BHI broth with added hemin and cultured overnight at 37°C, and an equal volume of the culture was mixed with the cryopreservation buffer and stored at −80°C for later use.

The remainder of the pure culture was centrifuged to collect the microbial biomass, which was then resuspended with a minimal volume of sterile Ringer’s solution. High-molecular-weight DNA was prepared from this biomass as described previously (6). Genome sequence data were produced using the Illumina NextSeq 500 system (2 × 150-bp high-output kit) with v2 chemistry and standardized protocols at the Australian Centre for Ecogenomics. The sequence data (150-bp paired-end sequence reads) were quality filtered using Trimmomatic v0.36 (7) and subjected to *de novo* assembly using the SPAdes Genome Assembler v3.11.0 (8); default parameters were used for all software described in this paper. The assembly consists of 78 contigs, with the largest one comprising 362,390 bp; a genome coverage of 290× was calculated using BamM v1.7.3 (http://ecogenomics.github.io/BamM/). The *N*_50_ and *L*_50_ values are 93,604 bp and 6 contigs, respectively. The estimated genome length is 2,053,207 bp, with a G+C content of 40%. The quality of the genome assembly was assessed using CheckM v1.1.3 (9) and estimated to be 99.9% complete and 0.15% contaminated. The taxonomic affiliation of the isolate was evaluated using both CheckM, RAST v2.0 (https://rast.nmpdr.org/), and GTDB-TK v1.5.0 (9–11), which all confirmed that the strain is a member of the Streptococcus salivarius lineage, now designated *S. salivarius* strain AGIRA0003. The AGIRA0003 draft genome sequence was aligned against the closest representative closed reference Streptococcus salivarius genome sequences (NCTC 8618, CCHSS3, and the genome submitted under GenBank accession number NZ_LR793266) to reorder the AGIRA0003 contigs before upload and annotation using the NCBI Prokaryotic Genome Annotation Pipeline (PGAP) v5.2. Finally, the plasmidVerify tool v1.0 (12) was used to examine the genome, but no evidence of plasmids was found.

### Data availability.

This whole-genome shotgun project has been deposited at DDBJ/ENA/GenBank under the accession number JAHCVC000000000. The version described in this paper is version number JAHCVC010000000. The raw sequence reads have been deposited under NCBI BioProject accession number PRJNA730991 and NCBI SRA data accession number SRX11549562. The *S. salivarius* AGIRA0003 culture is available from the National Measurement Institute (https://www.industry.gov.au/policies-and-initiatives/national-measurement-institute), submitted under the Budapest Treaty on the International Recognition of the Deposit of Microorganisms for the Purpose of Patent Procedure (accession number V21/008005).
